# Mammography with deep learning for breast cancer detection

**DOI:** 10.3389/fonc.2024.1281922

**Published:** 2024-02-12

**Authors:** Lulu Wang

**Affiliations:** Biomedical Device Innovation Center, Shenzhen Technology University, Shenzhen, China

**Keywords:** breast cancer, classification, X-ray mammography, artificial intelligence, machine learning, deep learning, medical imaging, radiology

## Abstract

X-ray mammography is currently considered the golden standard method for breast cancer screening, however, it has limitations in terms of sensitivity and specificity. With the rapid advancements in deep learning techniques, it is possible to customize mammography for each patient, providing more accurate information for risk assessment, prognosis, and treatment planning. This paper aims to study the recent achievements of deep learning-based mammography for breast cancer detection and classification. This review paper highlights the potential of deep learning-assisted X-ray mammography in improving the accuracy of breast cancer screening. While the potential benefits are clear, it is essential to address the challenges associated with implementing this technology in clinical settings. Future research should focus on refining deep learning algorithms, ensuring data privacy, improving model interpretability, and establishing generalizability to successfully integrate deep learning-assisted mammography into routine breast cancer screening programs. It is hoped that the research findings will assist investigators, engineers, and clinicians in developing more effective breast imaging tools that provide accurate diagnosis, sensitivity, and specificity for breast cancer.

## 1 Introduction

Breast cancer is one of the most prevalent cancers among females worldwide (1). Several factors, including gender, age, family history, obesity, and genetic mutations, contribute to the development of breast cancer (2). Early diagnosis with prompt treatment can significantly improve the 5-year survival rate of breast cancer (3). Medical imaging techniques like mammography and ultrasound are widely used for breast cancer detection (4, 5). Mammography utilizes low-dose X-rays to generate breast images that aid radiologists in identifying abnormalities like lumps, calcifications, and distortions (6). Mammography is recommended for women over 40, particularly those with a family history of breast cancer, as it effectively detects early-stage breast cancer (7). However, mammography has limitations, such as reduced sensitivity in women with dense breast tissue. To overcome these limitations, various imaging methods, such as digital breast tomosynthesis (DBT), ultrasound, magnetic resonance imaging (MRI), and positron emission tomography (PET), have been investigated as alternative tools for breast cancer screening.

DBT uses X-rays to generate three-dimensional breast images, which is particularly useful for detecting breast cancer in dense breasts (8). Compared to mammography, DBT provides higher accuracy and sensitivity in detecting breast cancer lesions. However, the interpretation of DBT images still faces inter-observer variability, which can affect its accuracy. Ultrasound imaging uses high-frequency sound waves to produce detailed images of breast tissue. Unlike mammography, ultrasound does not involve radiation, making it a safe method for detecting breast abnormalities, especially in women with dense breast tissue. Ultrasound helps evaluate abnormalities detected on a mammogram and can be used to monitor disease progression and assess treatment effectiveness (9). MRI has been recommended for women with high risks of breast cancer (10). PET utilizes a radioactive tracer to create breast images and is often used in conjunction with other imaging techniques, such as CT or MRI, to identify areas of cancer cells (11). Each of these imaging methods has its own set of advantages and disadvantages (12).

Artificial intelligence (AI) technologies have been extensively investigated to develop cancer prediction models (13, 14). AI-based models, such as machine learning (ML) algorithms, can analyze medical image datasets and patient characteristics to identify breast cancer or predict the risk of developing breast cancer. ML algorithms can extract quantitative features from medical images, such as mammograms or ultrasound images, through radiomics. AI-based prediction models can incorporate various cancer risk factors, including genetics, lifestyle, and environmental factors, to establish personalized imaging and treatment plans. In recent years, deep learning (DL) algorithms have emerged as promising AI tools to enhance the accuracy and efficiency of breast cancer detection (15). These data-driven techniques have the potential to revolutionize breast imaging by leveraging large amounts of data to automatically learn and identify complex patterns associated with malignancy.

This paper provides an overview of the recent developments in DL-based approaches and architectures used in mammography, along with their strengths and limitations. Additionally, the article highlights challenges and opportunities associated with integrating DL-based mammography to enhance breast cancer screening and diagnosis. The remaining sections of the paper are as follows: Section 2 describes the most popular medical imaging application for breast cancer detection. Section 3 discusses DL-based mammography techniques. Section 4 describes breast cancer prediction using DL techniques. Section 5 highlights the challenges and future research directions of DL approaches in mammography. Finally, Section 6 concludes the present study.

## 2 Medical imaging techniques for breast cancer detection

Medical imaging techniques have become essential in the diagnosis and management of breast cancer. This section provides an overview of several commonly used medical imaging techniques for breast cancer detection. **Table 1** compares the most widely utilized medical imaging methods for breast cancer.

**Table 1:** Comparison of medical imaging methods for breast cancer.

Reference	Techniques	Sensitivity	Tumor size corresponding to sensitivity	Advantages	Disadvantages
(16)	Mammography	85%	≤2 cm	Improved image resolution, widely available	Limited sensitivity in dense breast tissue, exposure to radiation
(17)	Ultrasound	82%	2 cm	No ionizing radiation, suitable for dense breasts and implant imaging	Operator-dependent, limited specificity
(18)	MRI	95%	≤2 cm	Images small details of soft tissues	Expensive
(19)	Diffused optical tomography	92.35%	1 cm	Non-invasive, safe	Illposed problem during reconstruction

### 2.1 Mammography

This section presents the working principle, recent advancements, advantages, and disadvantages of mammography. Mammography is a well-established imaging modality used for breast cancer screening. It is a non-invasive technique that utilizes low-dose X-rays to generate high-resolution images of breast tissue. Mammography operates based on the principle of differential X-ray attenuation. The breast tissue is compressed between two plates, and a low-dose X-ray beam is directed through the breast to create an image. Different types of breast tissues, such as fatty, glandular, and cancerous tissue, attenuate X-rays differently. The X-rays that pass through the breast tissue are detected by a digital detector, and an image of the breast is formed. The resulting image is a two-dimensional projection of the breast tissue. In recent years, mammography has undergone significant advancements. Digital mammography has replaced film-screen mammography, leading to improved image quality and reduced radiation dose. Digital breast tomosynthesis (DBT), a 3D mammography technique, has enhanced breast cancer detection rates and reduced false positives. Automated breast ultrasound (ABUS) is another imaging modality used in conjunction with mammography for breast screening, particularly in women with dense breast tissue.

Numerous studies have investigated the effectiveness of mammography for breast cancer screening, demonstrating that it can reduce breast cancer mortality rates, especially for women aged 50-74 years. Additional screening with MRI or ultrasound may be recommended for women with higher risk of breast cancer, such as those with a family history or genetic predisposition. Several leading companies and research groups have achieved significant advancements in the past decade. For example, Hologic’s Genius 3D mammography technology provides higher-resolution 3D images, increasing detection rates while reducing false positives (20). However, it entails higher radiation exposure and higher costs compared to traditional mammography.

Other developments include GE Healthcare and Siemens Healthineers’ contrast-enhanced spectral mammography (CESM), which combines mammography with contrast-enhanced imaging to improve diagnostic accuracy (21). Artificial intelligence tools developed by companies like iCAD and ScreenPoint Medical have been utilized to enhance mammography interpretation, leading to earlier breast cancer detection (22). Gamma Medica and Dilon Technologies have introduced new breast imaging technologies, such as molecular breast imaging and breast-specific gamma imaging, which utilize different types of radiation to provide more detailed images of breast tissue (23).

The University of Chicago has made strides in contrast-enhanced mammography (CEM), which is more accurate in detecting invasive breast cancers than traditional mammography alone. CEM provides detailed images of breast tissue without ionizing radiation, though it is not widely available and may not be covered by insurance (24). The Karolinska Institute’s work on breast tomosynthesis has shown that it is more sensitive in detecting breast cancer than traditional mammography. Tomosynthesis provides a 3D image of the breast, facilitating the detection of small tumors and reducing the need for additional imaging tests. However, it exposes patients to slightly more radiation, takes longer to perform, and is more expensive (25).

Mammography has certain limitations, including limited sensitivity in women with dense breast tissue, false positives leading to unnecessary procedures, radiation exposure that accumulates over time, inability to distinguish between benign and malignant lesions, inaccuracy in detecting small cancers or cancers in certain breast regions, and limited utility in detecting specific types of breast cancer, such as inflammatory breast cancer. To address these limitations, various new imaging technologies, such as DBT, ultrasound elastography, and molecular breast imaging, have been proposed and investigated. These technologies aim to provide more accurate and reliable breast cancer detection, particularly in high-risk individuals. Future research directions for mammography include improving test accuracy, utilizing AI for image interpretation, and developing new techniques utilizing different radiation or contrast agents.

### 2.2 Digital breast tomosynthesis

DBT was first introduced in the early 2000s. Unlike traditional Mammography, DBT can generate three-dimensional images, leading to more accurate breast cancer detection by reducing tissue overlap. DBT is particularly effective in detecting small tumors and reducing false positive results compared to mammography (26). Additionally, it exposes patients to less radiation. However, DBT is more expensive and may not be covered by insurance for all patients. It also requires specialized equipment and training for interpretation, which may not be widely available in all areas.

### 2.3 Ultrasound

Ultrasound imaging is a non-invasive, relatively low-cost imaging technique that does not involve exposure to ionizing radiation. It can be used as an adjunct to mammography for breast cancer screening, especially in women with dense breast tissue. Nakano et al. (27) developed real-time virtual sonography (RVS) for breast lesion detection. RVS combines the advantages of ultrasound and MRI and can provide real-time, highly accurate images of breast lesions. However, RVS requires specialized equipment and software, and its diagnostic accuracy may depend on the operator. Standardization of RVS protocols and operator training may improve its accuracy and accessibility.

Zhang et al. (28) conducted a study on a computer-aided diagnosis (CAD) system called BIRADS-SDL for breast cancer detection using ultrasound images. BIRADS-SDL was compared with conventional stacked convolutional auto-encoder (SCAE) and semi-supervised deep learning (SDL) methods using original images as inputs, as well as an SCAE using BIRADS-oriented feature maps (BFMs) as inputs. The experimental results showed that BIRADS-SDL performed the best among the four networks, with classification accuracy of around 92.00 ± 2.38% and 83.90 ± 3.81% on two datasets. These findings suggest that BIRADS-SDL could be a promising method for effective breast ultrasound lesion CAD, particularly with small datasets. CAD systems can enhance the accuracy and efficiency of breast cancer detection while reducing inter-operator variability. However, CAD systems may produce false-positive or false-negative results, and their diagnostic accuracy may depend on the quality of the input images. Integrating CAD systems with other imaging modalities and developing algorithms to account for image quality variations may improve their accuracy and reliability (29).

GE Healthcare (USA) developed the Invenia Automated Breast Ultrasound (ABUS) 2.0, which improves breast cancer detection, especially in women with dense breasts, by providing high-resolution 3D ultrasound images (30). Siemens Healthineers (Germany) developed the ACUSON S2000 Automated Breast Volume Scanner (ABVS), which also provides high-resolution 3D ultrasound images for accurate breast cancer detection, particularly in women with dense breasts (31). These automated systems enhance breast cancer detection rates, improve workflow, and reduce operator variability.

Canon Medical Systems (Japan) developed the Aplio i-series ultrasound system with the iBreast package, which offers high-resolution breast imaging, leading to improved diagnostic performance for breast cancer detection. Invenia ABUS 2.0 and ACUSON S2000 ABVS are automated systems, while Aplio i-series with iBreast package requires manual scanning. The advantages of ABUS 2.0 and ACUSON S2000 ABVS include enhanced image quality, improved workflow, and reduced operator variability. However, they are more expensive than traditional mammography, and image interpretation may be time-consuming. Ultimately, the choice of system depends on the needs and preferences of healthcare providers and patients. Future research is likely to focus on improving the accuracy of ultrasound imaging techniques, developing new methods for detecting small calcifications, and reducing false-positive results.

### 2.4 Magnetic resonance imaging

MRI utilizes strong magnetic fields and radio waves to generate images of the body’s internal structures, making it one of the most important diagnostic tools. It has various applications, including the diagnosis and monitoring of neurological, musculoskeletal, cardiovascular, and oncological conditions. Its ability to image soft tissues makes it well-suited for breast imaging. Breast MRI is a non-invasive technique used for the detection and monitoring of breast cancer. It is often used in conjunction with mammography and ultrasound to provide a comprehensive evaluation of breast tissue.

Kuhl et al. (32) were the first to investigate post-contrast subtracted images and maximum-intensity projection for breast cancer screening with MRI. This approach offers advantages in terms of speed, cost-effectiveness, and patient accessibility. However, abbreviated MRI has limitations, including lower specificity and the potential for false positives. Mann et al. (33) studied ultrafast dynamic contrast-enhanced MRI for assessing lesion enhancement patterns. The use of new MRI sequences and image reconstruction techniques improved the specificity in distinguishing between malignant and benign lesions. Zhang et al. (34) explored a deep learning-based segmentation technique for breast MRI, which demonstrated accurate and consistent segmentation of breast regions. However, this method has limitations, such as its reliance on training data and potential misclassification.

MRI has several advantages, including the absence of ionizing radiation and increased accuracy in detecting small tumors within dense breast tissue. However, it is expensive, time-consuming, and associated with a higher false-positive rate. Future research directions involve developing faster and more efficient MRI techniques and utilizing AI techniques to enhance image analysis and interpretation.

**Figure 1:**
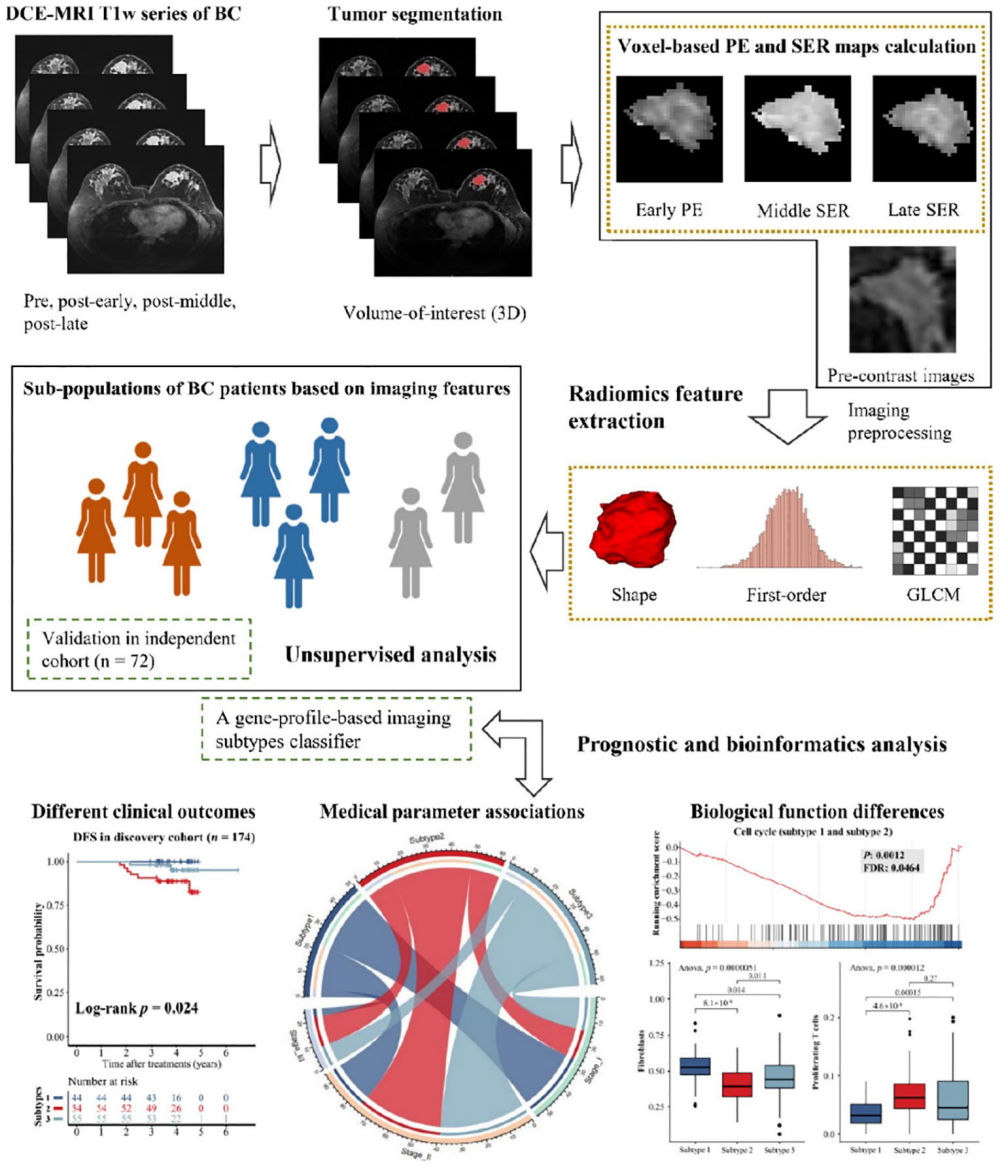
Workflow of unsupervised analysis based on DCE-MRI features in breast cancer patients (35).

Contrast-enhanced MRI (DCE-MRI) has recently become a crucial method in clinical practice for the detection and evaluation of breast cancer. **Figure 1** illustrates the workflow of unsupervised analysis based on DCE-MRI radiomics features in breast cancer patients (35). Ming et al. (35) utilized DCE-MRI to calculate voxel-based percentage enhancement (PE) and signal enhancement ratio (SER) maps of each breast. This study collected two independent radiogenomics cohorts (n = 246) to identify and validate imaging subtypes. The results demonstrated that these imaging subtypes, with distinct clinical and molecular characteristics, were reliable, reproducible, and valuable for non-invasive prediction of the outcome and biological functions of breast cancer.

### 2.5 Positron emission tomography

PET is an advanced imaging technique that has made significant contributions to the diagnosis and treatment of breast cancer. It is a non-invasive procedure that provides healthcare professionals with valuable information about the spread of cancer to other parts of the body, making it an essential tool in the fight against breast cancer. With ongoing technological advancements, PET plays a crucial role in the detection and treatment of breast cancer.

PET utilizes radiotracers to generate three-dimensional images of the interior of the body. It operates by detecting pairs of gamma rays emitted by the radiotracer as it decays within the body. PET imaging was first introduced in the early1950s, and the first PET scanner was developed in the 1970s. Since then, PET has become an indispensable tool for cancer detection. It has been commonly used to diagnose and stage cancer and assess the effectiveness of cancer treatments. It is also utilized in cardiology, neurology, breast, and psychiatry.

PET is more sensitive than mammography and ultrasound in detecting small breast tumors, and it can also distinguish between benign and malignant lesions with higher accuracy (36). The advantages of PET include its non-invasive and safety for repeated use. However, PET does have limitations, including limited availability, higher cost compared to mammography and ultrasound, a higher rate of false positives, and the requirement for radiotracer injection.

## 3 Deep learning-based mammography techniques

Several DL architectures, including convolutional neural networks (CNN), transfer learning (TL), ensemble learning (EL), and attention-based methods, have been developed for various applications in mammography. These applications include breast cancer detection, classification, segmentation, image restoration and enhancement, and computer-aided diagnosis (CAD) systems.

CNN is an artificial neural network with impressive results in image recognition tasks. CNN recognizes image patterns using convolutional layers that apply filters to the input image. The filters extract features from the input image, passing through fully connected layers to classify the image. Several CNN-based methods have been proposed in mammography for breast tumor detection. Wang et al. (37) applied CNN with transfer learning in ultrasound for breast cancer classification. The proposed method achieved an area under the curve (AUC) value of 0.9468 with five-folder cross-validation, for which the sensitivity and specificity were 0.886 and 0.876, respectively. Shen et al. (38) proposed a deep CNN in Mammography to classify benign and malignant and achieved an accuracy of 0.88, higher than radiologists (0.83). The study showed that CNN had a lower false-positive rate than radiologists. Yala et al. (39) developed CNN-based mammography to classify mammograms as low or high risk for breast cancer and achieved an AUC of 0.84, which was higher than that of radiologists (0.77). CNN had a lower false-positive rate than radiologists, which has shown promising results in improving the accuracy of mammography screening. CNN has several advantages over traditional mammography screening, including higher accuracy, faster processing, and the ability to identify subtle changes in mammograms. CNN requires large amounts of data to train the network and may not be able to detect all types of breast cancer. Further research is needed to investigate the use of CNN in Mammography.

CNN is an artificial neural network that has shown impressive results in image recognition tasks. It recognizes image patterns using convolutional layers that apply filters to the input image. These filters extract features from the input image, which then pass through fully connected layers to classify the image. In Mammography, several CNN-based methods, such as DenseNet, ResNet, and VGGNet, have been proposed for breast tumor detection. For example, Wang et al. (37) applied CNN with transfer learning in ultrasound for breast cancer classification, achieving an area under the curve (AUC) value of 0.9468 with five-fold cross-validation. The sensitivity and specificity were 0.886 and 0.876, respectively. Shen et al. (38) proposed a deep CNN in mammography to classify between benign and malignant tumors, achieving an accuracy of 0.88, higher than that of radiologists (0.83). Yala et al. (39) developed a CNN-based mammography system to classify mammograms as low or high risk for breast cancer, achieving an AUC of 0.84, higher than that of radiologists (0.77). These studies demonstrated that CNN had a lower false-positive rate than radiologists, showing promise in improving the accuracy of mammography screening. CNN offers advantages over traditional mammography screening, including higher accuracy, faster processing, and the ability to identify subtle changes in mammograms. However, CNN requires large amounts of data to train the network and may not be able to detect all types of breast cancer. Further research is needed to investigate the use of CNN in mammography.

TL utilizes pre-trained DL models to train on small datasets. TL-based methods have shown promising results in improving the accuracy of mammography for breast tumor detection. EL combines multiple DL models to improve the accuracy of predictions. EL-based approaches, such as stacking, boosting, and bagging, have been proposed in mammography for breast tumor detection.

Attention-based methods use attention mechanisms to focus on critical features of the image. Several attention-based methods, such as SE-Net and Channel Attention Networks (CAN), have been proposed for breast tumor detection in mammography. DL is a type of ML that uses neural networks to learn and make predictions. DL methods have gained popularity in recent years due to their ability to work with large datasets and extract meaningful patterns and insights.

DL methods have revolutionized the field of machine learning and are being used in an increasing number of applications, ranging from self-driving cars to medical imaging. As datasets and computing power continue to grow, these methods are expected to become even more powerful and prevalent in the future.

## 4 Breast cancer prediction using deep learning

This section presents the recent developments in DL methods for breast cancer prediction. The DL-based breast cancer prediction techniques involves the following steps:

- Data Collection: Breast datasets are obtained from various sources such as medical institutions, public repositories, and research studies. These datasets consist of mammogram images, gene expression profiles, and clinical data.
- Data Preprocessing: The collected datasets are preprocessed to eliminate noise, normalize, and standardize the data. This step involves data cleaning, feature extraction, and data augmentation.
- Model Building: DL models, such as CNNs, RNNs, DBNs, and autoencoders, are developed using the preprocessed breast cancer datasets. These models are trained and optimized using training and validation datasets.
- Model Evaluation: The trained DL models are assessed using a separate test dataset to determine their performance. Performance metrics, including sensitivity, specificity, accuracy, precision, F1 score, and AUC, are used for evaluation.
- Model Interpretation: The interpretability of the DL models is evaluated using techniques such as Grad-CAM, saliency maps, and feature visualization. These techniques help identify which features of the input data are utilized by the DL models for making predictions.
- Deployment: The DL model is deployed in a clinical setting to predict breast cancer in patients. The performance of the model is regularly monitored and updated to enhance accuracy and efficiency.

By utilizing DL techniques, breast cancer prediction can be significantly improved, leading to better detection and treatment outcomes.

### 4.1 Data preprocessing techniques and evaluation

#### 4.1.1 Preprocessing techniques

When applying DL algorithms to analyze breast images, noise can have a negative impact on the accuracy of the image classifier. To address this issue, several image denoising techniques have been developed. These techniques, including the Median filter, Wiener filter, Non-local means filter, Total variation (TV) denoising, Wavelet-based denoising, Gaussian filter, anisotropic diffusion, BM3D denoising, CNN, and autoencoder, aim to reduce image noise while preserving important features and structures that are relevant for breast cancer diagnosis.

After denoising, a normalization method, such as min-max normalization, is typically employed to rescale the images and reduce the complexity of the image datasets before feeding them into the DL model. This normalization process ensures that the model can effectively learn meaningful patterns from the images and improve its ability to accurately classify them.

#### 4.1.2 Performance metrics

Several performance metrics are utilized to evaluate DL algorithms for breast screening. The selection of a specific metric depends on the task at hand and the objectives of the model. Some of the most commonly employed metrics include:

- Accuracy: measures the proportion of correct predictions made by the model.
- Precision: measures the proportion of true positive predictions out of all positive predictions made by the model.
- Sensitivity: measures the proportion of true positive predictions out of all actual positive cases in the dataset.
- F1 score: a composite metric that balances precision and sensitivity.
- Area under the curve (AUC): distinguishes between positive and negative points across a range of threshold values.
- Mean Squared Error (MSE): measures the average squared difference between predicted and actual values in a regression task.
- Mean Absolute Error (MAE): measures the average absolute difference between the predicted and actual values in a regression task.

The commonly used equation for calculating accuracy, as stated in reference (40), is:


(1) $$\text{Accuracy}=\frac{\text{TP}+\text{TN}}{\text{TP}+\text{TN}+\text{FP}+\text{FN}}$$

Where TP and TN are the numbers of true positives and true negatives, FP and FN are the numbers of false positives and false negatives, respectively.


(2) $$\text{Precision}=\frac{\text{TP}}{\text{TP}+\text{FP}}$$

(3) $$\text{Sensitivity}=\frac{\text{TP}}{\text{TP}+\text{FN}}\;$$

(4) $$\text{F}1\text{ score}=\frac{2*(\text{Precision }*\text{ Sensitivity})}{\text{Precision}+\text{Sensitivity}}$$

AUC is typically computed by plotting the true positive rate against the false positive rate at different threshold values and then calculating the area under this curve.


(5) $$\text{MSE}=(1/\text{n})*{\sum \left(y_{true}-y_{pred}\right)}^{2}\;$$

(6) $$\text{MAE}=(1/\text{n})*\sum \left|y_{true}-y_{pred}\right|$$

Where *y_tru_* is the true value and *y_pred_* is the predicted value, and n is the number of samples.

**Equations 1**–**6** provide a general idea of how performance metrics are computed, but the actual implementation may vary depending on the specific task and the software.

### 4.2 Datasets

Breast datasets play a crucial role in evaluating DL approaches. These datasets offer a comprehensive collection of high-quality and labelled breast images that can be utilized for training and testing DL algorithms. **Table 2** presents commonly utilized publicly available breast datasets in mammography for breast screening.

**Table 2:** Breast image dataset.

Year	Country	Dataset	Sample Number	Human Number	Task
1998 (41)	US	Digital Database for Screening Mammography (DDSM)	2,620	N/A	Breast cancer detection
1998 (42)	US	Mammographic Image Analysis Society (Mini-MIAS)	322	N/A	Breast cancer classification
2012 (43)	US	INbreast	410	N/A	Breast cancer detection
2017 (44)	US	Breast Cancer Digital Repository (BCDR)	1,224	N/A	Breast cancer classification
2017 (45)	US	Curated Breast Imaging Subset of DDSM (CBIS-DDSM)	753	N/A	Breast cancer detection
2011 (46)	US	BCDR-F01	362	N/A	Breast cancer classification
2018 (47)	USA	DDSM	2,620	N/A	Classification, segmentation
2016 (48)	Netherlands	Mammographic Image Analysis Society (MIAS)	322	N/A	Classification, segmentation
2012 (49)	Multi-country	BCDR	1,875	N/A	Classification, density

N/A, Not Applicate.

### 4.3 Breast lesion segmentation

The Nottingham Histological Grading (NHG) system is currently the most commonly utilized tool for assessing the aggressiveness of breast cancer (50). According to this system, breast cancer scores are determined based on three significant factors: tubule formation (51), nuclear pleomorphism (52), and mitotic count (53). Tubule formation is an essential assessment factor in the NHG grading system for understanding the level of cancer. Before identifying tubule formation, detection or segmentation tasks need to be performed. Pathologists typically conduct these tasks visually by examining whole slide images (WSIs). Medical image segmentation assists pathologists in focusing on specific regions of interest in WSIs and extracting detailed information for diagnosis. Conventional and AI methods have been applied in medical image segmentation, utilizing handcrafted features such as color, shapes, and texture (54–56). Traditional manual tubule detection and segmentation techniques have been employed in medical images. However, these methods are challenging, prone to errors, exhaustive, and time-consuming (57, 58).

**Table 3** provides a comparison of recently developed DL methods in mammography for breast lesion segmentation. These methods include the Conditional Random Field model (CRF) (59), Adversarial Deep Structured Net (60), Deep Learning using You-Only-Look-Once (61), Conditional Residual U-Net (CRU-Net) (62), Mixed-Supervision-Guided (MS-ResCU-Net) and Residual-Aided Classification U-Net Model (ResCU-Net) (63), Dense U-Net with Attention Gates (AGs) (64), Residual Attention U-Net Model (RU-Net) (65), Modified U-Net (66), Mask RCNN (67), Full-Resolution Convolutional Network (FrCN) (68), U-Net (69), Conditional Generative Adversarial Networks (cGAN) (70, 71), DeepLab (72), Attention-Guided Dense-Upsampling Network (AUNet) (73), FPN (74), modified CNN based on U-Net Model (76), deeply supervised U-Net (77), modified U-Net (78), and Tubule-U-Net (79). Among these DL methods, U-Net is the most commonly employed segmentation method.

**Table 3:** Deep learning approaches in mammography for breast lesion segmentation.

Year	Model	Evaluation Dataset	Noise remove method	Performance Metrics (results)
2015 (59)	CRF	INbreast and DDSM-BCRP	NA	The method achieved an 89.0% Dice index in 0.1.
2018 (60)	adversarial deep structured net	INbreast and DDSM-BCRP	NA	The method achieved a segmentation rate of 97.0%.
2018 (61)	deep learning using You-Only-Look-Once	INbreast	NA	The method achieved detection rate of 98.96%, Matthews correlation coefficient (MCC) of 97.62%, and F1 score of 99.24%.
2018 (62)	CRU-Net	INbreast and DDSM-BCRP	NA	The CRU-Net achieved a Dice Index DI of 93.66% for INbreast and a DI of 93.32% for DDSM-BCRP.
2019 (63)	MS-ResCU-Net and ResCU-Net	INbreast	NA	The MS-ResCU-Net achieved an accuracy of 94.16%, sensitivity of 93.11%, specificity of 95.02%, DI of 91.78%, Jac of 85.13%, and MCC of 87.22%, while ResCU-Net correspondingly achieved 92.91%, 91.51%, 94.64%, 90.50%, 83.02%, and 84.99%.
2019 (64)	dense U-Net with AGs	DDSM	NA	The method achieved 82.24% F1 score, 77.89% sensitivity, and overall accuracy of 78.38%.
2019 (65)	RU-Net	DDSM, BCDR-01, and INbreast	cLare filter	The proposed model achieved a mean test pixel accuracy of 98.00%, a mean Dice coefficient index (DI) of 98.00%, and mean IOU of 94.00%.
2019 (66)	modified U-Net	DDSM	Laplacian filter	The method produced 98.50% of the F-measure and a 97.80% Dice score, Jaccard index of 97.40%, and average accuracy of 98.20%.
2020 (67)	mammographic CAD based on pseudocolour mammograms and mask RCNN	INbreast	morphological filters	The DSI achieved for mass segmentation was 0.88Â ± 0.10, and GMs and mask RCNN yielded an average TPR of 0.90Â ± 0.05.
2020 (68)	FrCN	INbreast	NA	FrCN achieved an overall accuracy of 92.97%, 85.93% for MCC, 92.69% for Dice, and 86.37% for the Jaccard similarity coefficient.
2020 (69)	U-Net	CBIS-DDSM, INbreast, UCHCDM, and BCDR-01	adaptive median filter	The U-Net model achieved a mean Dice coefficient index of 95.10% and a mean IOU of 90.90%.
2020 (70)	cGAN	INbreast	median filter	The cGAN achieved an accuracy of 98.0%, Dice coefficient of 88.0%, and Jaccard index of 78.0%.
2020 (71)	cGAN	DDSM and INbreast	Morphological operations	The proposed cGAN model achieved a Dice coefficient of 94.0% and an intersection over union (IoU) of 87.0%
2020 (72)	mask RCNN and DeepLab	MIAS and DDSM	Savitzky Golay filter	The mask RCNN achieved an AUC of 98.00%, DeepLab achieved an AUC of 95.00%.
2020 (73)	AUNet	CBIS-DDSM and INbreast	NA	produced an average Dice similarity coefficient of 81.80% for CBIS-DDSM and 79.10% for INbreast
2020 (74)	mask RCNN-FPN	training on DDSM and testing on the INbreast database	NA	The model achieved a mean average precision of 84.0% for multidetection and 91.0% segmentation accuracy.
2020 (75)	U-Net	DDSM	NA	The model achieved a sensitivity of 92.32%, specificity of 80.47%, accuracy of 85.95%, Dice coefficient index of 79.39%, and AUC of 86.40%.
2021 (76)	modified CNN based on U-Net model	DDSM-400 and CBIS-DDSM	NA	The method achieved a diagnostic performance of 89.8% and AUC of 86.20% based on ground-truth segmentation maps and a maximum of 88.0% and 86.0% for U-Net-based segmentation for DDSM-400 and CBIS-DDSM, respectively.
2021 (77)	deeply supervised U-Net	DDSM and INbreast	cLare filter	The method achieved 82.70% of Dice, 85.70% of Jaccard coefficient, 99.70% accuracy, 83.10% sensitivity, and 99.80% specificity.
2021 (78)	modified U-Net	MIAS, DDSM, and CBIS-DDSM	NA	The method achieved accuracy of 98.87%, AUC of 98.88%, sensitivity of 98.98%, precision of 98.79%, and F1 score of 97.99% on the DDSM datasets
2023 (79)	Tubule-U-Net	30820 polygonal annotated tubules in 8225 patches	NA	achieved 95.33%, 93.74%, and 90.02%, dice, sensitivity, and specificity scores, respectively

N/A, Not Applicate.

Naik et al. (80) developed a likelihood method for the segmentation of lumen, cytoplasm, and nuclei based on a constraint: a lumen area must be surrounded by cytoplasm and a ring of nuclei to form a tubule. Tutac et al. (81) introduced a knowledge-guided semantic indexing technique and symbolic rules for the segmentation of tubules based on lumen and nuclei. Basavanhally et al. (82) developed the O’Callaghan neighborhood method for tubule detection, allowing for the characterization of tubules with multiple attributes. The process was tested on 1226 potential lumen areas from 14 patients and achieved an accuracy of 89% for tubule detection. In reference (83), the authors applied a k-means clustering algorithm to cluster pixels of nuclei and lumens. They employed a level-set method to segment the boundaries of the nuclei surrounding the lumen, achieving an accuracy of 90% for tubule detection. Romo-Bucheli et al. (84) developed a Convolutional Neural Network (CNN) based detection and classification method to improve the accuracy of nuclei detection in tubules, achieving an accuracy of 90% for tubule nuclei detection. Hu et al. (85) proposed a breast mass segmentation technique using a full CNN (FCNN), which showed promising results with high accuracy and speed. Abdelhafiz et al. (86) studied the application of deep CNN for mass segmentation in mammograms and found increased performance in terms of accuracy. Tan et al. (87) recently developed a tubule segmentation method that investigates geometrical patterns and regularity measurements in tubule and non-tubule regions. This method is based on handcrafted features and conventional segmentation techniques, which are not effective and efficient for tubule structures due to their complex, irregular shapes and orientations with weak boundaries.

### 4.4 Deep learning approaches in mammography for breast lesion detection and classification

DL approaches have garnered considerable attention in mammography for the detection and classification of breast lesions, primarily due to their ability to automatically extract high-level features from medical images. Numerous popular DL algorithms have been employed in mammography for breast screening, including convolutional neural networks (CNN), deep belief networks (DBN), recurrent neural networks (RNN), autoencoders, generative adversarial networks (GAN), capsule networks (CN), convolutional recurrent neural networks (CRNN), attention mechanisms, multiscale CNN, and ensemble learning (EL).

CNN proves highly effective in extracting and classifying image features into distinct categories. DBN is particularly advantageous in identifying subtle changes in images that may be challenging for human observers to discern. RNN utilizes feedback loops to facilitate predictions, thereby aiding in the analysis of sequential data. Autoencoders are utilized for unsupervised feature learning, which aids in the detection and classification of mammography images. GAN is exceptionally effective in generating synthetic mammography images for training DL models. CN is highly proficient in detecting and classifying mammography images. CRNN combines CNN and RNN, making it particularly useful in analyzing sequential data. Attention mechanisms focus on specific areas of mammography images, proving beneficial in detecting and classifying images that encompass intricate structures and patterns. Multiscale CNN analyzes images at multiple scales, proving invaluable in detecting and classifying images with complex structures and patterns at varying scales. EL combines multiple DL models to enhance accuracy and reduce false positives.

**Table 4** analyzes the recently developed DL methods for breast lesion detection using mammography. These methods have the potential to greatly enhance the accuracy and efficiency of breast cancer diagnosis. However, it is important to note that most DL methods for biomedical imaging applications come with certain limitations. These limitations include the need for large training datasets, being limited to mass spectrometry images, and being computationally expensive.

**Table 4:** DL-based mammography for breast tumor detection.

Reference	Year	Method	Database	Number of images	Accuracy	AUC	Sensitivity	Specificity
(88)	2016	Deep CNN	DDSM	600	96.7%	NA	NA	NA
(89)	2016	AlexNet	FFDM	607	NA	86%	NA	NA
(90)	2016	CNN	BCDR-F03	736	NA	82%	NA	NA
(91)	2016	SNN	UCI, DDSM	NA	89.175%, 86%	NA	NA	NA
(92)	2016	ML-NN	ED(US)	NA	98.98%	98%	NA	NA
(93)	2016	DBN	ED(US-SW E)	NA	93.4%	94.7%	88.6%	97.1%
(94)	2017	Deep CNN	FFDM	3185	82%	88%	81%	72%
(95)	2017	CNN (COM)	INbreast	115	95%	91%	NA	NA
(96)	2017	Deep CNN	SFM, DM	2242	NA	82%	NA	NA
(97)	2017	CNN-CT	IRMA	2796	83.74%	83.9%	79.7%	85.4%
(97)	2017	CNN-WT	IRMA	2796	81.83%	83.9%	78.2%	83.3%
(98)	2017	VGG19	FFDM	245	NA	86%	NA	NA
(99)	2017	Custom CNN	FFDM	560	NA	79%	NA	NA
(100)	2017	VGG16	IRMA	2795	100%	100%	NA	NA
(101)	2017	SNN	DDSM	480	79.5%	NA	NA	NA
(101)	2017	CNN (COM)	MIAS, CBIS-INBreast	NA	57%	77%	NA	NA
(96)	2017	Multitask DNN	ED(Mg),DD SM	1057 malignant, 1397 benign	82%	NA	NA	NA
(102)	2017	CNN (COM)	ED (HP)	NA	95.9% (2 classes), 96.4% (15 classes)	NA	NA	NA
(103)	2017	ImageNet	BreakHis	NA	93.2%	NA	NA	NA
(104)	2018	GoogLeNet	BCDR-F03	736	81%	88%	NA	NA
(104)	2018	AlexNet	BCDR-F03	736	83%	79%	NA	NA
(104)	2018	Shallow CNN	BCDR-F03	736	73%	82%	NA	NA
(105)	2018	Faster R-CNN	INbreast	115	NA	95%	NA	NA
(105)	2018	Faster R-CNN	DREAM	82,000	NA	85%	NA	NA
(106)	2018	ROI based CNN	DDSM	600	97%	NA	NA	NA
(107)	2018	Inception V3	DDSM	5316	97.35% ( ± 0.80)	98%	NA	NA
(107)	2018	Inception V3	INbreast	200	95.50% ( ± 2.00)	97%	NA	NA
(107)	2018	Inception V3	BCDR-F03	600	96.67% ( ± 0.85)	96%	NA	NA
(107)	2018	VGG16	DDSM	5316	97.12% ( ± 0.30)	NA	NA	NA
(107)	2018	ResNet50	DDSM	5316	97.27% ( ± 0.34)	NA	NA	NA
(108)	2018	Deep CNN	MIAS	120	96.7%	NA	NA	NA
(109)	2018	AlexNet, Transfer Learning	University of Pittsburgh	20,000	NA	98.82%	NA	NA
(110)	2018	Faster R-CNN	DDSM, INbreast & Semmelweis University data	2620,115, 847	NA	95%	NA	NA
(111)	2018	CNN	FFDM	78	NA	81%	NA	NA
(112)	2018	MV-DNN	BCDR-F03	736	85.2%	89.1%	NA	NA
(113)	2018	Deep CNN	MIAS	322	65%	NA	NA	NA
(114)	2018	SDAE	ED (HP)	58	98.27% (Benign), 90.54% (Malignant)	NA	97.92% (Benign), 90.17% (Malignant)	NA
(115)	2018	CNN (UDM)	BreakHis	NA	96.15%, 98.33% (2 Classes), 83.31-88.23% (8 Classes)	NA	NA	NA
(116)	2018	CNN-CH	BreakHis	400× (× represents magnificati on factor)	96%	NA	97.79%	90.16%
(116)	2018	CNN-CH	BreakHis	400× (× represents magnificati on factor)	97.19%	NA	98.20%	94.94%
(117)	2019	CNN	DDSM	190	93.24%	NA	91.92%	91.92%
(117)	2019	CNN based LBP	DDSM	190	96.32%	97%	96.81%	95.83%
(118)	2020	InceptionV3	DDSM	2620	79.6%	NA	89.1%	NA
(118)	2020	ResNet 50	DDSM	2620	85.7%	NA	87.3%	NA
(119)	2020	ResNet50	DDSM patch	10713	75.1%	NA	NA	NA
(119)	2020	Mobile Net	DDSM patch	10713	77.2%	NA	NA	NA
(119)	2020	MVGG16	DDSM patch	10713	80.8%	NA	NA	NA
(119)	2020	MVGG16 + ImageNet	DDSM patch	10713	88.3%	93.3%	NA	NA
(71)	2020	GAN and CNN	DDSM	292	80%	80%	NA	NA
(120)	2021	Optimal Multi-Level Thresholding-based Segmentation with DL enabled Capsule Network (OMLTS-DLCN)	Mini-MIAS dataset and DDSM dataset	NA	98.5% for Mini-MIAS, 97.55% for DDSM	NA	NA	NA
(121)	2021	Inception-ResNet-V2	BreastScreen Victoria dataset	28,694	0.8178	0.8979	NA	NA
(122)	2021	AI-powered imaging biomarker		2,058	NA	0.852	NA	NA
(123)	2022	DualCoreNet	DDSM	NA	NA	0.85	NA	NA
(123)	2022	DualCoreNet	INbreast	NA	NA	0.93	NA	NA

N/A, Not Applicate.

**Table 5** presents a comprehensive list of the latest DL-based mammogram models developed for breast lesion classification. DL models offer numerous benefits, including exceptional accuracy and optimal performance achieved with fewer parameters. However, it is important to acknowledge certain limitations associated with existing DL methods for breast tumor classification using mammographies. These limitations include the substantial computational power and extensive datasets required for training the models, which can be computationally expensive, intricate, and time-consuming.

**Table 5:** DL-based mammography for breast tumor classification.

Reference	Year	Method	Database	Number of images	Accuracy	AUC	Sensitivity	Precision	F1-Score
(95)	2017	Transfer learning, Random Forest	INbreast	108	90%	NA	98%	70%	NA
(124)	2018	Deep GeneRAtive Multitask	CBIS-DDSM	NA	89%	0.884	NA	NA	NA
(125)	2019	VGG, Residual Network	CBIS-DDSM	NA	NA	NA	86.10%	80.10%	NA
(126)	2019	DCNN, Alexnet	CBIS-DDSM	1696	75.0%	0.80	NA	NA	NA
(127)	2019	MA-CNN	MIAS	322	96.47%	0.99	96.00%	NA	NA
(128)	2019	DCNN, MSVM	MIAS	322	96.90%	0.99	NA	NA	NA
(129)	2019	CNN Improvement (CNNI-BCC)	MIAS	NA	90.50%	0.90	89.47%	90.71%	NA
(130)	2020	MobileNet, VGG, Resnet, Xception	CBIS-DDSM	1696	84.4%	0.84	NA	NA	85.0%
(131)	2020	MobilenetV1, MobilenetV2	CBIS-DDSM	1696	74.5%	NA	NA	70.00%	76.00%
(132)	2020	DE-Ada*	CBIS-DDSM	NA	87.05%	0.9219	NA	NA	NA
(133)	2020	AlexNet	MIAS	68	98.53%	0.98	100%	97.37%	98.3%
(133)	2020	GoogleNet	MIAS	68	88.24%	0.94	80%	94.74%	85.71%
(134)	2020	Inception ResNet V2	INbreast	107	95.32%	0.95	NA	NA	NA
(132)	2020	De-ada*	INbreast	NA	87.93%	0.9265	NA	NA	NA
(135)	2021	CNN	CBIS-DDSM	1592	91.2%	0.92	92.31%	90.00%	91.76%
(136)	2021	MobilenetV2, Nasnet Mobile, MEWOM	CBIS-DDSM	1696	93.8%	0.98	93.75%	93.80%	93.77%
(137)	2021	ResNet-18, (ICS-ELM)	MIAS	322	98.13%	NA	NA	NA	NA
(135)	2021	CNN	MIAS	322	93.39%	0.94	92.72%	94.12%	93.58%
(136)	2021	Mobilenet V2 & NasNet Mobile, MEWOA	MIAS	300	99.80%	1.00	99.00%	99.33%	99.16%
(137)	2021	ResNet-18, (ICS-ELM)	INbreast	179	98.26%	NA	NA	NA	NA
(135)	2021	CNN	INbreast	387	93.04%	0.94	94.83%	91.23%	93.22%
(136)	2021	Fine-tuned MobilenetV2, Nasnet, MEWOM	INbreast	108	99.7%	1.00	99.0%	99.0%	99.0%
(123)	2022	DualCoreNet	CBIS-DDSM	NA	NA	0.85± 0.021	NA	NA	NA
(138)	2022	CNN classifier with different fine-tuning	DDSM	13128	99.96%	1.00	100%	99.92%	99.96%

N/A, Not Applicate.

## 5 Challenges and future research directions

The emergence of DL techniques has revolutionized medical imaging, offering immense potential to enhance the diagnosis and treatment of various diseases. DL algorithms present several advantages compared to traditional ML methods. For instance, DL algorithms can be trained using robust hardware such as graphical processing units (GPU) and tensor processing units (TPU), greatly accelerating the training process. This has enabled researchers to train large DL models with billions of parameters, yielding impressive results in diverse language tasks. However, to fully leverage the potential of DL in medical imaging, several challenges must be addressed. One of the primary challenges is the scarcity of data. DL algorithms require abundant, high-quality data for effective training. Yet, acquiring medical imaging data is often challenging, particularly for rare diseases or cases requiring long-term follow-up. Furthermore, data privacy regulations and concerns can further complicate the availability of medical imaging data. Another challenge lies in the quality of annotations. DL algorithms typically demand substantial amounts of annotated data for effective training. However, annotating medical imaging data can be subjective and time-consuming, leading to issues with annotation quality and consistency. This can significantly impact the performance of deep learning algorithms, particularly when accurate annotations are vital for diagnosing or treating specific conditions. Additionally, imbalanced classes pose another challenge in medical imaging.

In numerous instances, the occurrence of certain states may be relatively low, which can result in imbalanced datasets that have a detrimental effect on the performance of DL algorithms. This situation can pose a significant challenge, especially for rare diseases or conditions with limited data availability. Another crucial concern in medical imaging is the interpretability of models. Although DL algorithms have showcased remarkable performance across various medical imaging tasks, the lack of interpretability in these models can hinder their adoption. Clinicians frequently necessitate explanations for the predictions made by these algorithms in order to make informed decisions, but the opacity of DL models can make this task arduous.

Data privacy is a paramount concern in medical imaging. Medical images encompass confidential patient information, stringent regulations dictate the utilization and dissemination of such data. The effective training of DL necessitates substantial access to extensive medical imaging data, thereby introducing challenges concerning data privacy and security. Additionally, computational resources pose another challenge in the realm of medical imaging. DL algorithms mandate substantial computational resources for the effective training and of models. This predicament can prove particularly troublesome in medical imaging, given the size and intricacy of medical images, which can strain computing resources. DL algorithms can be vulnerable to adversarial attacks, where small perturbations to input data can cause significant changes in the model’s output. This can be particularly problematic for medical imaging, where even small changes to an image can have substantial implications for diagnosis and treatment.

Several potential strategies can be employed to address these challenges effectively. One approach involves the development of transfer learning techniques, enabling DL models to be trained on smaller datasets by leveraging information from related tasks or domains. This approach holds particular promise in medical imaging, where data scarcity poses a significant obstacle. Another approach involves placing emphasis on the development of annotation tools and frameworks that enhance the quality and consistency of annotations. This becomes important in cases where annotations play a critical role in diagnosing or treating specific conditions. Furthermore, improved data sharing and collaboration between institutions can help alleviate both data scarcity and privacy concerns. By pooling resources and sharing data, it becomes feasible to construct more extensive and diverse datasets that can be employed to train DL models with greater effectiveness. Additionally, enhancing the interpretability of DL models in medical imaging techniques stands as another critical area of research. The development of explainable AI techniques can provide clinicians with valuable insights into the underlying factors contributing to a model’s predictions. Lastly, bolstering the robustness of DL models constitutes a crucial focal point. This entails exploring adversarial training techniques, as well as leveraging ensemble methods and other strategies to enhance the overall robustness and generalizability of DL models.

DL techniques have the potential to revolutionize medical imaging. However, to fully leverage this potential, it is crucial to address several challenges. These challenges encompass data scarcity, annotation quality, imbalanced classes, model interpretability, data privacy, computational resources, and algorithm robustness. By prioritizing strategies to tackle these challenges, it becomes possible to develop DL models that are more effective and reliable for various medical imaging applications.

## 6 Conclusion

This paper examines the recent advancements in DL-based mammography for breast cancer screening. The authors have investigated the potential of DL techniques in enhancing the accuracy and efficiency of mammography. Additionally, they address the challenges that need to be overcome for the successful adoption of DL techniques in clinical practice.

## Author contributions

LW: Conceptualization, Data curation, Formal analysis, Funding acquisition, Investigation, Methodology, Project administration, Resources, Software, Visualization, Writing – original draft, Writing – review & editing.
